# Blue Lasing at Room Temperature Based on a Quasi-Bound State in the Continuum

**DOI:** 10.1021/acs.nanolett.6c01930

**Published:** 2026-07-11

**Authors:** Tomasz Fąs, Emilia Pruszyńska-Karbownik, Marta Sawicka, Dmitriy Yavorskiy, Anna Feduniewicz, Oliwia Gołyga, Mateusz Słowikowski, Jacek Kacperski, Marcin Siekacz, Grzegorz Muzioł, Piotr Nowicki, Aleksandr Kazakov, Jerzy Wróbel, Tomasz Czyszanowski, Jan Suffczyński

**Affiliations:** † Institute of Experimental Physics, Faculty of Physics, University of Warsaw, 5 Pasteura St., 02-093 Warsaw, Poland; ‡ Institute of High Pressure Physics, Polish Academy of Sciences, 29/37 Sokolowska St., 01-142 Warsaw, Poland; § CENTERA laboratories, Institute of High Pressure Physics, Polish Academy of Sciences, 29/37 Sokolowska St., 01-142 Warsaw, Poland; ∥ CENTERA, CEZAMAT, Warsaw University of Technology, 19 Poleczki St., 02-822 Warsaw, Poland; ⊥ CEZAMAT, Warsaw University of Technology, 19 Poleczki St., 02-822 Warsaw, Poland; # Institute of Physics, Polish Academy of Sciences, 32/46 Lotnikow Av., 02-668 Warsaw, Poland; 7 International Research Centre MagTop, Institute of Physics, Polish Academy of Sciences, 32/46 Lotnikow Av., 02-668 Warsaw, Poland; 8 Institute of Physics, Łódź University of Technology, 217/221 Wólczańska St., 90-451 Łódź, Poland

**Keywords:** bound state in continuum, gallium nitride, subwavelength grating, lasing, photoluminescence, nanoporous GaN

## Abstract

We demonstrate room-temperature lasing in the blue spectral range, 440 to 460 nm, where optical gain is provided by InGaN quantum wells coupled to quasi-bound states in the continuum hosted by a monolithic GaN-based subwavelength grating. A low-refractive-index nanoporous GaN layer obtained by electrochemical etching, placed beneath the structure, suppresses the coupling of the optical modes to the substrate. The gratings are designed by theoretical modeling and fabricated with sub-10 nm precision using e-beam lithography and dry etching. The presence of quasi-bound states in the continuum is confirmed by angle-resolved reflectivity and observation of a polarization vortex. The structures ensure a low threshold (0.16 mJ cm^–2^ pulse^–1^), narrow spectral bandwidth (below 0.04 nm), and strongly linearly polarized lasing emission. The efficient tunability of the lasing wavelength is achieved by adjusting the grating geometry parameters. This work opens new opportunities for compact and tunable monolithic GaN-based photonic devices.

## Graphical abstract

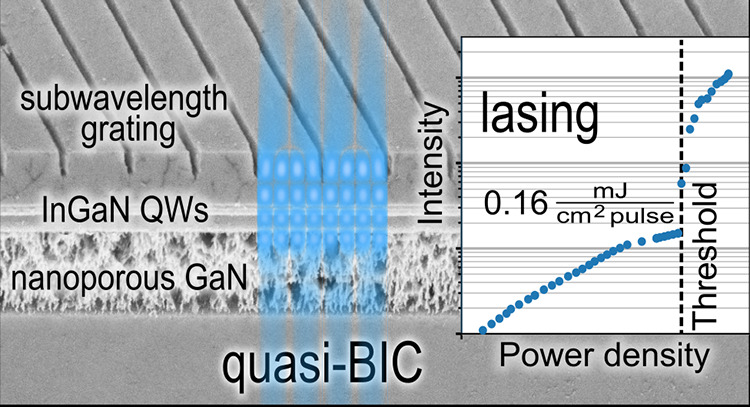

Bound state in the continuum (BIC) represents a peculiar type of an optical mode that, despite existing within the radiation continuum, remains nonradiative due to symmetry protection or topological constraints. Although an infinite quality factor (Q-factor) is a property of an ideal BIC, in practice BICs exhibit very high, but finite Q-factors, while still enabling efficient light confinement and enhanced light–matter interactions for applications in lasing devices and optical sensing.

Among different types of photonic structures hosting BICs,^1^ a 1D periodic structure in the form of a subwavelength grating (SWG) is particularly attractive. The SWG is a diffraction grating with a period smaller than the wavelength of incident light. The SWG distinguishes itself among photonic structures by, e.g., simplicity of the design and fabrication as well as polarization selectivity.

The efficiency of light confinement in the SWG layer and the associated suppression of radiative losses increase with the refractive index difference between the grating and the substrate on which it is deposited.^2^ To achieve the largest refractive index contrast, the SWG typically takes the form of a suspended membrane^3^ or requires the integration of the semiconductor with a low-refractive-index dielectric, which complicates the growth and deteriorates the performance of the device. Here, we choose gallium nitride (GaN) as the grating material, and to increase the refractive index contrast and thereby decouple the SWG optical modes from the substrate in a fully monolithic structure that can be realized within a single epitaxial growth process, we introduce a low-refractive-index porous GaN layer beneath the grating. GaN offers numerous advantages for visible photonics, including a wide bandgap of 3.4 eV at 300 K, which eliminates interband absorption in the visible spectral range, good thermal transport, and resistance to oxidation and humidity. The refractive index of GaN around 2.3 at 500 nm^4^ provides a sufficient contrast with air.^5^

While the epitaxial growth of GaN is well established, the performance of III-nitride-based optical components, such as GaN/(Al,Ga)N distributed Bragg reflectors, is constrained by the relatively small refractive index contrast within the III-nitride material system. In turn, integration of GaN with materials offering a higher refractive index contrast is hindered by large lattice mismatch, resulting in strain relaxation and defect formation, thereby degrading optical performance. Previous approaches to GaN-based SWGs have been limited mostly to GaN-based suspended membranes incorporating a high-contrast grating reflector.^6–9^ Independently of the material system, in approaches combining quantum wells (QWs) and the SWG so far, distributed Bragg reflectors (DBRs) placed underneath the QW layers were employed to mitigate the leakage of the optical mode to the volume of the substrate.^3,10,11^

In the present work, we present an epitaxially grown monolithic structure, in which (In,Ga)­N/GaN QWs serving as the gain medium are inserted between the GaN-based SWG and the nanoporous GaN layer. Porous GaN emerged recently as an attractive material in the III-nitride material system because of the advantageous tuning of its refractive index with a degree of porosity in a range that is inaccessible for bulk (In,Al,Ga)N alloys (Figure S1).^12,13^ A successful implementation of nanoporous GaN in highly reflective DBRs,^12,14^ vertical-cavity surface-emitting lasers,^15^ and its application as a cladding material for edge-emitting laser diodes has already been reported.^16–18^ The porous GaN layer provides a strain-free mechanical support^19^ and, owing to its significantly lower refractive index compared to GaN prevents leakage of the optical mode of the SWG into the substrate, without the need to use a multilayer DBR. The use of epitaxially grown (In,Ga)­N/GaN QWs ensures emission in the blue–green spectral range and enables wavelength tuning via adjustment of QW thickness and/or indium composition. This approach contrasts with previous works, in which a single GaN layer simultaneously served as both the SWG material and the gain medium.^20^ Here, we design the SWGs through theoretical analysis and experimentally demonstrate the BIC states confined by them. The fabricated structures exhibit low-threshold lasing at 0.16 mJ · cm^–2^ per pulse, with spectrally and angularly narrow emission and a high degree of linear polarization.

We design and study two types of structures: a passive structure, with a subwavelength GaN grating lying directly on a nanoporous GaN layer, and an active structure with (In,Ga)­N/GaN QWs additionally placed between the SWG and the nanoporous layer (Figures 1 and 2). The passive structure enables the determination of the achievable quality factor of the optical mode hosting a quasi-BIC in a structure with nanoporous GaN serving as a low-refractive-index cladding. The active structure allows us to demonstrate room-temperature lasing in the blue spectral region based on the quasi-BIC with (In,Ga)­N/GaN QWs being the gain medium.

**Figure 1:**
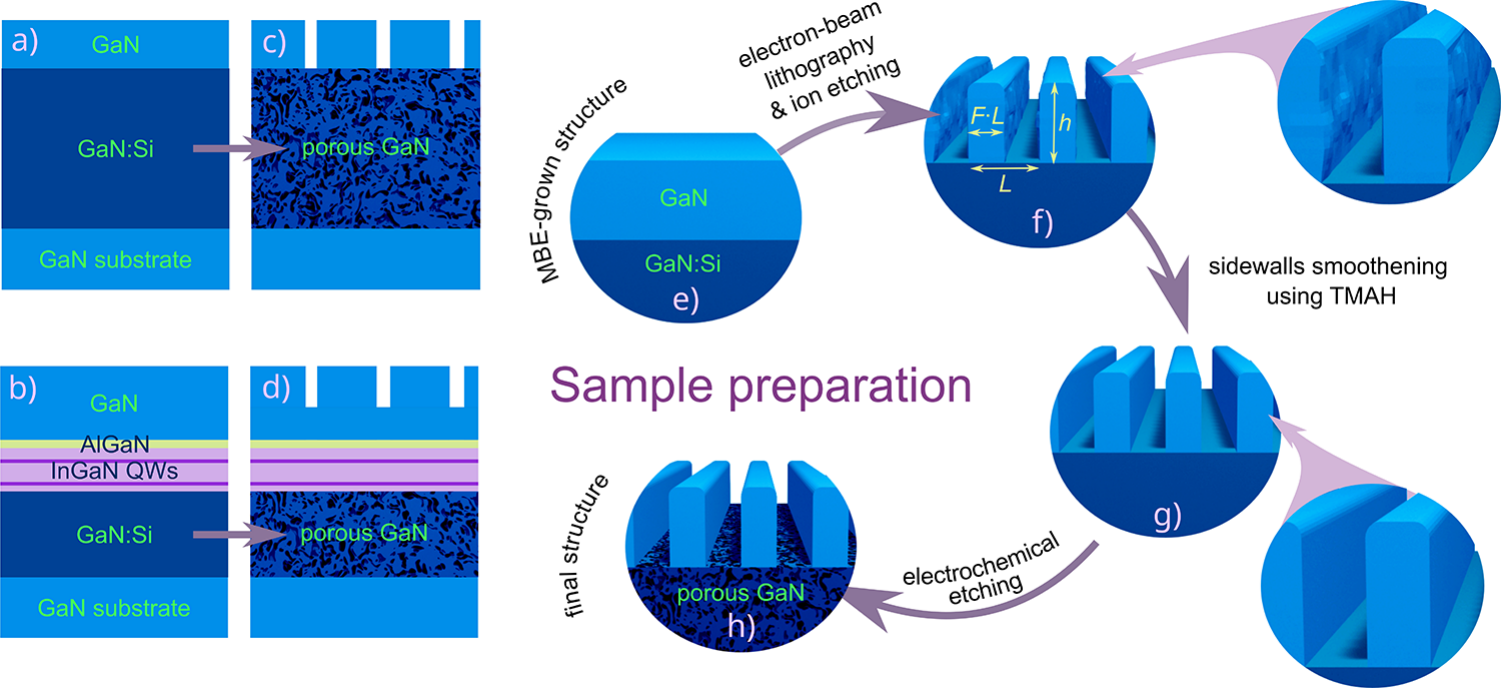
A schematic cross-section of an epitaxial “as-grown”: (a) passive structure and (b) active structure. A cross-section of a final (c) passive structure and (d) active structure. Scheme of the sample processing, taking as an example the passive structure: (e) the cross-section of the epitaxially grown structure, (f) electron-beam lithography and reactive ion etching of the subwavelength grating, (g) optional smoothening of the grating sidewalls in the TMAH solution, and (h) the final structure after electrochemical etching that induced formation of the nanoporous GaN layer.

**Figure 2:**
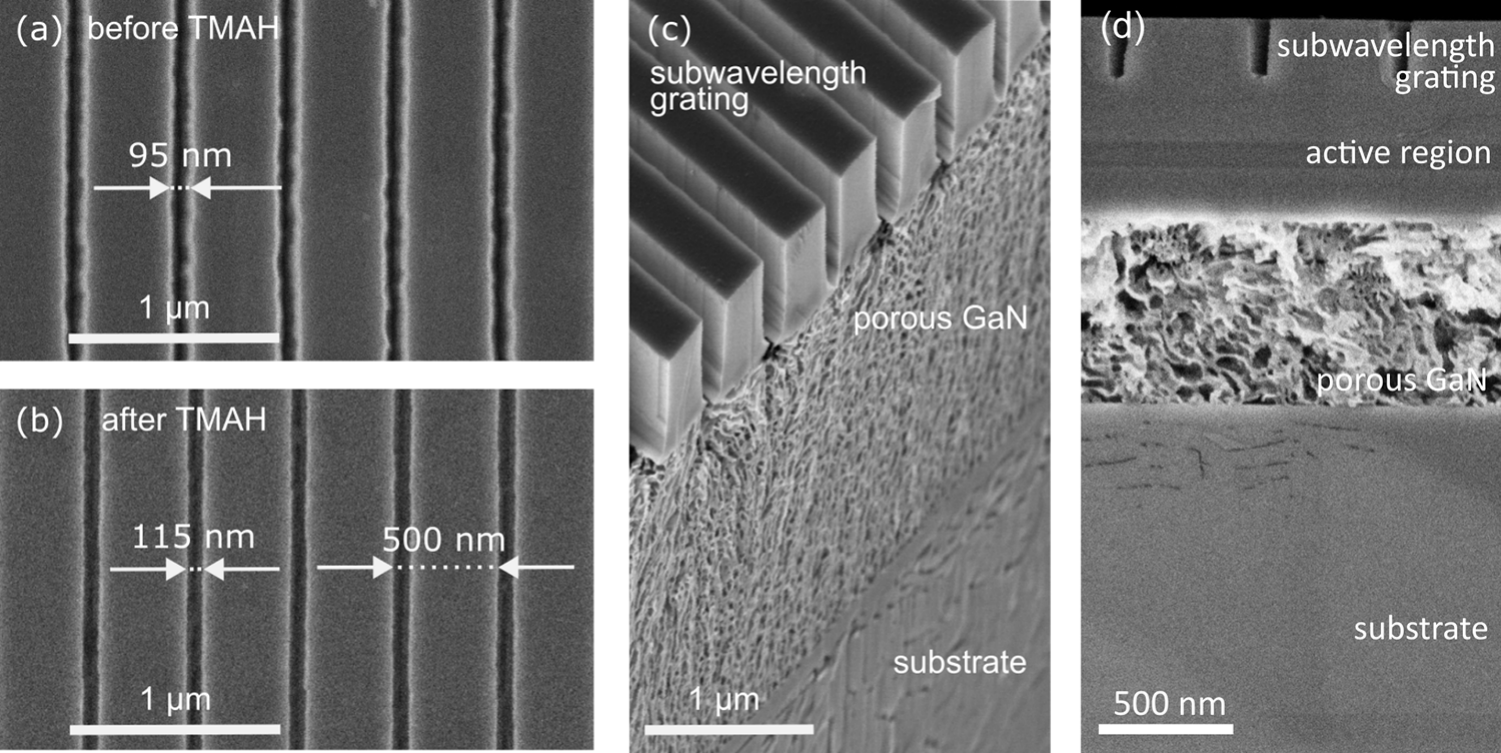
SEM images of the passive structure (a) before and (b) after TMAH treatment, viewed from the top, showing the smoothening effect of the subwavelength grating sidewalls. (c) SEM image of a subwavelength grating on porous GaN viewed from the side after cleaving across the passive structure. (d) SEM image of a subwavelength grating on nanoporous GaN with (In,Ga)­N/GaN quantum wells in the active structure, viewed from the side.

The grating parameters that ensure light confinement in the form of BIC states are determined through a systematic numerical investigation using the plane-wave admittance method (PWAM).^21^ We consider the SWG of height *h* of up to 10 μm, the periodicity *L* = 500 nm, the wavelength λ of the light incident on the structure from the air in the range of 350 to 1150 nm, nanoporous GaN layer of thickness *h*_*p*_ in the range from 0 to 2 μm and the porosity *F*_*P*_ from 0 (i.e., solid GaN) to 1 (i.e., air). We consider the ratio of the stripe width to the grating period (that is, the fill factor *F*), which varies from 0.1 to 0.9.

In the analysis, we determine the resonant wavelength of antisymmetric modes, potentially forming symmetry-protected BICs,^1^ and map them as a function of *L* and *h* normalized with respect to the wavelength (see Figure S2a). Here, the modes are labeled with names, where the first letter indicates symmetry (S for symmetric, A for antisymmetric), while the first digit denotes the number of horizontal nodal points within a single SWG stripe, and the second digit denotes the number of vertical nodal points across the structure. The antisymmetric nature of the modes is verified by checking whether the calculated electric field distribution within the grating period exhibits minima along both symmetry axes of the subwavelength grating, located at the center and at the boundary of the stripe, as shown in Figure 3d. As our calculations show, for the SWG periods smaller than 0.65λ, the calculated modes are symmetry-protected BICs (modes A20 – A22 in Figure S2). This value corresponds to the subwavelength condition in the nanoporous layer λ/*n*_*P*_, where *n*_*P*_ ∼ 1.5 is the refractive index of the nanoporous GaN with assumed porosity of *F_P_* = 0.75. For the periods exceeding λ/*n*_*P*_ ∼ 0.65λ, the first order of diffraction into the substrate becomes allowed. In such a case, optical modes can form interference-based BICs with an infinite quality factor^22^ or collapse into quasi-BICs (modes A40–A44 in Figure 3b and Figure S2). We define a quasi-BIC as an optical mode that originates from an ideal bound state in the continuum but exhibits a finite, albeit very high, quality factor due to symmetry breaking. In a finite structure, any BIC collapses into a quasi-BIC as a result of its finite size. Furthermore, as discussed in the experimental section, the *Q* factor of such modes is additionally reduced by imperfections in periodicity arising from fabrication inaccuracies, as well as by material absorption.

**Figure 3:**
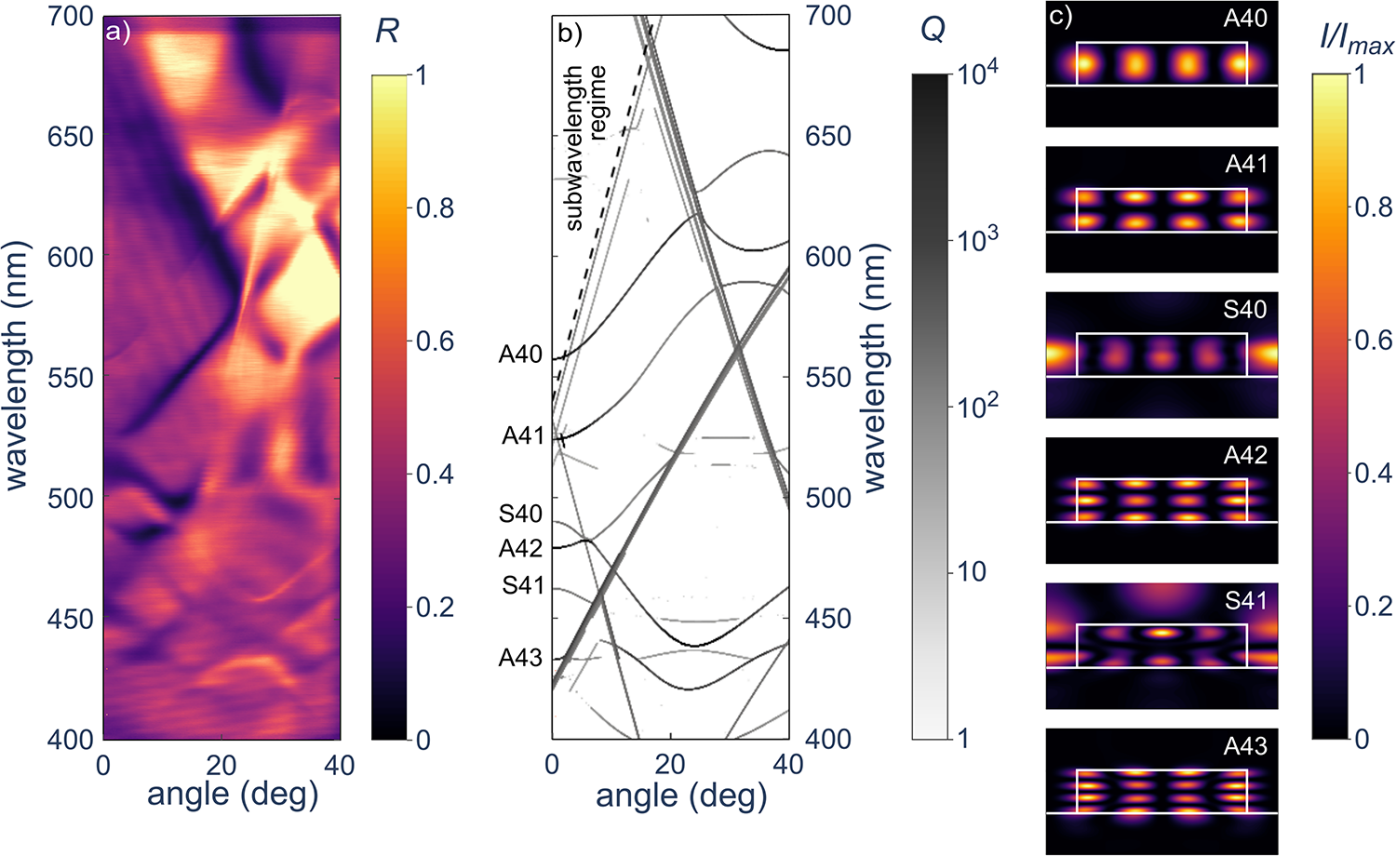
(a) Experimentally obtained angle-resolved reflectivity map of the passive structure in TE polarization and (b) numerically calculated dispersion curves of the SWG modes. The color intensity of the curves encodes the *Q*-factor value. A black dashed lines indicate the cutoff of the first diffraction order of the grating. (c) Cross sections of the numerically calculated light intensity profiles in a SWG stripe for the antisymmetric (A40, A41, A42, A43) and symmetric (S40, S41) modes, whose dispersion curves are marked in panel (b). The calculations are performed for the structure with the parameters determined from SEM images: period *L* = 540 nm, stripe height *h* = 505 nm, fill factor *F* = 0.7354, and porosity *F*_*P*_ = 0.67. White lines indicate the boundaries between materials: GaN, nanoporous GaN, and air above the structure.

In addition, the calculations of the quality factor as a function of the nanoporous GaN thickness *h*_*P*_, conducted for porosity of F_p_ = 0.67, indicate that BICs can be present in the structure if the *h*_*P*_ exceeds 1.5 μm, while quasi-BICs obtain their maximum *Q* values for *h*_*P*_ of the order of several hundred nanometers and more (see Figure S2b).

The samples are grown by plasma-assisted molecular beam epitaxy, as this technique ensures high interface quality and enables efficient *n*-type doping of GaN, even as high as 1 · 10^20^ cm^–3^, which is required for efficient porosification of the layers, while maintaining high structural quality and surface smoothness.^23,24^ Both studied structures comprise an undoped top GaN layer on a Si-doped GaN layer deposited on a GaN substrate. In the case of the active structure, two 10.4 nm wide (In,Ga)­N/GaN quantum wells are additionally introduced between the undoped GaN and the GaN:Si layer. The QWs are located at the antinodes of the anticipated optical quasi-BIC mode A43. Mode A43 is selected as it ensures equally strong spatial resonance with both QW layers. A schematic cross-section of as-grown passive and active structures is shown schematically in Figure 1a and b. The Table S1 and Table S2 summarize the passive and active design of the epitaxial structures, respectively.

The sample processing technology developed in this work enables the monolithic fabrication of high-quality GaN-based SWGs covering an area of 100 × 100 μm^2^ on porous GaN, characterized by a rectangular profile and very smooth stripe edges, thus with properties improved with respect to previous approaches.^6–9,25–27^ The fabrication steps of the studied samples are presented schematically in Figure 1e–g. The electron beam lithography process, followed by the reactive ion etching (Figure 1e) performed on the “as-grown” structure results in the SWG formed out of the top GaN layer, as shown in Figure 1f. The SWG period *L*, height *h*, and fill factor *F* are denoted in the schematics. Next, the sample is treated with a TMAH solution to improve the sidewalls smoothness, as depicted in Figure 1g and highlighted in the insets. The last step is electrochemical etching, which induces a controllable formation of the nanoporous structure within the GaN:Si layer (Figure 1h). In the case of the passive structure, the etching proceeds vertically from the top surface of the sample. In the case of the active structure, the etching develops in a horizontal direction after the introduction of additional grooves, granting access to the GaN:Si layer, as described in ref.^24^

The samples are grown according to the theoretical design. The SWG period *L* in the fabricated passive structure is varied from 440 to 500 nm, and groove widths are varied between 65 and 190 nm in steps of (10 ± 3) nm, keeping the constant stripe height of (505 ± 5) nm. The *L* in the active structure is varied from 440 to 470 nm, and three groove widths are (75 ± 3) nm, (85 ± 3) nm and (95 ± 3) nm with the corresponding stripe heights *h* (225 ± 10) nm, (250 ± 10) nm, and (275 ± 10) nm. The thickness of the nanoporous GaN layer is *h*_*P*_ = 2 μm in the passive structure and *h*_*P*_ = 600 nm in the active structure.

Arrays of SWGs have been fabricated in active and passive structures. The SEM images of the exemplary SWG are presented in Figure 2 and in Figure S6. The smoothening effect of the TMAH treatment is directly reflected by the comparison of the top view of the passive structure presented in Figure 2a and b. We estimate the decrease in sidewall roughness and changes in groove widths by image processing of top-view SEM images. We used an edge-detection algorithm based on detecting the maxima and minima in an image’s exposure. As a result of the TMAH treatment, the calculated mean root-mean square roughness parameter *R*_*q*_ decreases from (4.76 ± 0.15) nm to (3.73 ± 0.12) nm. The width of the groove increases on average by (22.5 ± 0.5) nm, while the grating period *L* remains unchanged. Changes in roughness and width are determined for gratings with groove widths in the range from 130 to 180 nm, and, in this range, they do not depend on the groove widths. Results of numerical simulations indicate that such improvement in wall smoothness increases the *Q* factor of the considered modes by a factor of 1.6 (see Figure S5).

The schematic cross sections of the final passive and active structures with a porous GaN layer beneath the SWG are shown in Figure 1c and d, respectively. Additional optical and electron microscopy images of the active structure are shown in Figure S6.

Section S1 in the Supporting Information provides details about methods of calculations and structure design, while details of the geometry, epitaxy, and processing are provided in Section S2.

The angle-resolved reflectivity map of a SWG on the passive structure registered in TE polarization of the signal is shown in Figure 3a (the angle represents photon in-plane momentum in the *k*_*x*_ direction, see the inset to Figure S7). A rich modal structure of optical modes is visible. To distinguish the SWG-confined modes from waveguide modes in the nanoporous layer, we perform additional calculations considering a structure incorporating a GaN-based nanoporous layer deposited on a GaN substrate, but with a layer of material with the effective refractive index of the SWG replacing the SWG. In that way, we find that the modes with a nonmonotonic dispersion and a Fano-like shape in Figure 3a are the modes of the SWG. Selected SWG modes are indicated in Figure 3b. The mode labeled A40 has a wavelength longer than the SWG period and thus remains in the subwavelength regime. The mode vanishes for the zero angle in the reflectivity map (that is, Γ-point of the photon in-plane momentum), indicating BIC or quasi-BIC presence. The calculated quality factor of the A40 mode is 2 · 10^3^, which identifies it as a quasi-BIC. Additional confirmation of the presence of quasi-BIC states in the studied structures is provided by polarization-resolved reflectivity measurements, which reveal polarization vortices associated with the quasi-BIC. An example vortex in the vicinity of the quasi-BIC state in the passive structure is shown in Figure S8.

As our calculations reveal, the majority of the modes with a monotonic angular dependence (visible as a weak, background grid in the reflectivity map and in the simulation’s results shown in Figure S3b) are associated with light confinement in the nanoporous GaN layer.

An exemplary angle-resolved emission spectrum below the lasing threshold of the active structure registered at room temperature in TE polarization is presented in Figure 4a. Apparently, emission via the SWG modes dominates, while the leaky modes propagating in the nanoporous layer do not appear in the spectrum. There is a quasi-BIC state at around 445 nm centered at *k* = 0 (direction normal to the grating plane), identified by numerical calculation as A43 mode. The absence of emission at *k* = 0 indicates the expected decoupling of the quasi-BIC from the air, preventing it from accepting or emitting light. Above the threshold (see Figure 4b), the emission takes the form of spectrally and angularly very narrow spots, and is limited solely to the vicinity of the quasi-BIC state. To determine the emission parameters as a function of excitation power, the emission is integrated over a narrow angular range (−3° to +3°) for sequentially acquired PL maps. Next, the intensity, center, and full width at half-maximum (FWHM) of the emission is determined. The onset of the lasing action is confirmed by the strong nonlinear increase in the emission intensity above a threshold value of 0.16 mJ · cm^–2^ per pulse, accompanied by a blue-shift and significant narrowing of the emission (see Figure 4c and d). The FWHM of the emission attains 0.04 nm, reaching the spectral resolution of the experimental setup. Still, 0.04 nm is a record-low value for a single-mode, GaN-based near-UV laser of any type, and for any lasing based on a BIC state reported so far.^20^ Emission spectra of the active structure for consecutively increasing excitation power density are shown in Figure S9.

**Figure 4:**
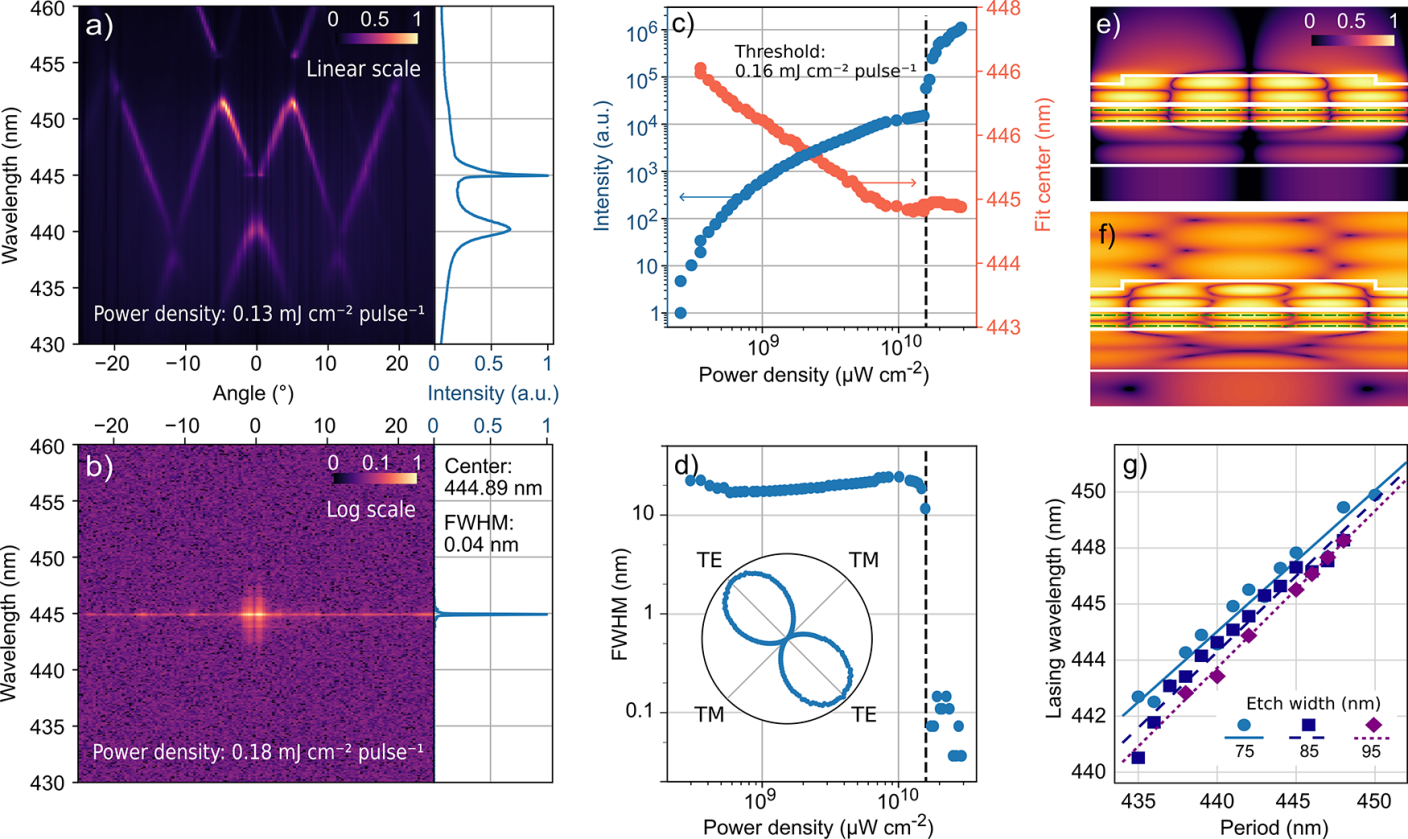
Angle resolved photoluminescence of the active structure incorporating (In,Ga)N quantum wells coupled to a quasi-BIC mode confined in a GaN-based subwavelength grating (a) below and (b) above the lasing threshold. The map in (b) is shown in a logarithmic scale for clarity. The inset shows the zoom of the emission spectrum above the lasing threshold. A frequency-doubled femtosecond laser delivers pulsed excitation at 375 nm. Parameters of the structure: period *L* = 439 nm, depth of etching *h* = 284 nm, width of etched stripes *d* = *L*(1 – *F*) = 75 nm. (c) Integrated emission intensity and spectral position, as well as (d) full width at half-maximum (FWHM) of the spectra as a function of the excitation power. The uncertainty of the FWHM value determination is 0.05 nm. Inset to (d): Intensity of emission as a function of the detected linear polarization angle, evidencing a strong linear (TE) polarization of the lasing. Cross sections of the numerically calculated light intensity profiles in a SWG stripe for (e) the antisymmetric A43 mode at λ ∼ 445 nm that supports lasing and (f) the symmetric S43 mode at λ ∼ 440 nm, both present in the map shown in panel (a). White lines indicate the boundaries between materials, while green dashed lines indicate positions of the InGaN QWs. (g) Wavelength of the quasi-BIC based lasing as a function of the SWG period *L* determined from the experiment for structures with etch width *d* varied from 75 to 95 nm with corresponding SWG heights of *h* = 225, 250, and 275 nm for *d* = 75, 85, and 95 nm, respectively. The solid lines represent a linear regression. A clear trend in the experimental points is evidenced.

In the reflectivity measurement two polarizations are present in the incident beam, hence both, TE and TM polarized, components may be, in principle, present in the reflected signal. In particular, in Figure S8, we see that depending on the projection of the reflected-light wavevector in the vicinity of the BIC state onto the grating plane, the reflected light is either fully TE or TM polarized (horizontal or vertical directions of the plot), or both polarization components contribute to the signal (intermediate directions). We deal with a different situation in the emission. Here, a high quality TE polarized optical mode hosting BIC and supporting the lasing to a large extent imposes the polarization of the emitted signal. Consequently, for wavevectors at which a TM polarized reflectivity signal is registered in reflectivity, the corresponding emission signal is practically absent. In the case of the studied structure, TE polarized lasing emission is 52 times stronger than the TM polarized one; see the inset to Figure 4d. Once we perform a polarization resolved measurement in photoluminescence, such as in reflectivity, to trace the linear polarization rotation around the lasing at around 445 nm, we see that the linear polarization of the signal rotates around a Γ-point also in the emission (see Figure S11), corroborating the fact that the emission takes place from the vicinity of a BIC state. We note, however, that the angular distribution of the emission shown in Figure S11 is the same for TE and for both diagonal polarizations, with the emission slightly tilted from the normal due to a nonzero component of the wavevector perpendicular to the stripes. It changes to a perpendicular spatial direction only for a strictly TM-polarized signal. Hence, although the rotation of the polarization is observable in the lasing emission, the emission is predominantly TE polarized, and its far field direction remains strongly pinned to the direction defined by the reduced rotational symmetry of the SWG.

The polarization anisotropy of the emission is observed already below the lasing threshold, as reflected by the TE-polarized signal being around 4 times stronger than the TM-polarized signal, see Figure S10a. The anisotropy results from the polarization selectivity of the SWG, in which the electric field oscillating along the stripes defines a preferred (TE) polarization direction. The increase of the polarization anisotropy of emission for around an order of magnitude, reflected by the ratio of TE- to TM-polarized signal attaining 52, is explained by the pinning of the lasing mode to the TE-polarized mode of the SWG. Factors such as nonzero birefringence of the nanoporous layer or anisotropy related to the crystallographic axes of the GaN host are not meaningful, as the polarization of the emission in the case of each of the studied gratings is defined solely by the grating geometry.

The numerically calculated cross sections of the light intensity profiles in a SWG stripe for the antisymmetric A43 mode at λ ∼ 445 nm, which supports quasi-BIC-based lasing, are shown in Figure 4e. It confirms a very good confinement of light within the stripe volume. In the case of the symmetric S43 mode at λ ∼ 440 nm (see Figure 4f), the confinement of light is much weaker, with an expected stronger effect of the mode leakage to the surroundings.

Despite the SWG fabrication process, which involves several steps, including electron beam lithography, etching, and TMAH smoothing, the gratings maintain the intended parameters. Variation in the SWG period and groove width with sub-10 nm spatial resolution allows us to study the impact of geometric parameters on grating performance. The lasing is observed in most of the fabricated SWGs, and the resulting statistics of the lasing wavelength are shown in Figure 4g. In addition, as Figure S10b–d shows, parameters of the emission do not vary meaningfully when probing different spatial positions within a given grating. We have checked that the decrease in temperature from 300 to 8 K lowers the lasing threshold only slightly, decreasing its value by a factor of 1.5. This indicates that temperature-activated nonradiative processes are not meaningful in the structures studied.

With the present work, we show the application of quasi-bound states in the continuum hosted by a gallium nitride-based subwavelength grating for room-temperature lasing in the blue spectral range. Innovative monolithic structures incorporating GaN-based subwavelength gratings have been designed by systematic theoretical modeling. A low-refractive-index porous GaN layer placed beneath the SWG efficiently mitigates leakage of optical modes into the GaN substrate, thereby replacing a commonly used multilayered DBR.

The lasing shows a low threshold (0.16 mJ cm^–2^ pulse^–1^), record-narrow line width of the emission line as for GaN-based lasing structures (0.04 nm), and strongly linearly polarized emission. We prove the efficient tunability of the lasing wavelength by varying the grating geometry parameters. In addition, the use of indium–gallium nitride quantum wells coupled to the quasi-BIC is expected to enable advantageous tuning of the emission via the In content or the QW width, in contrast to previous approaches in which GaN layers served as the gain medium.

## Supplementary Material



## Notes

Numerical simulations and design of the subwavelength grating structures; refractive index dispersion curves for GaN, nanoporous GaN, AlGaN, and InGaN; fabrication details including epitaxial growth and processing; reflectivity measurement setup and polarization vortex data; excitation density and polarization-dependent photoluminescence; additional SEM images (PDF)
