# Supplementary material for: Blue Lasing at Room Temperature Based on a Quasi-Bound State in the Continuum
Source: Nano Lett. 2026 Jul 11;26(28):9170–6. doi: 10.1021/acs.nanolett.6c01930 (PMC13397883; doi:10.1021/acs.nanolett.6c01930)
Supplement: Supplementary file 1 [file nl6c01930_si_001.pdf]

Supporting Information to "Blue lasing at room temperature based on a quasi-bound state in the continuum"

Tomasz Fąs[1], Emilia Pruszyńska-Karbownik[1], Marta Sawicka[2], Dmitriy Yavorskiy[3,4], Anna Feduniewicz[2], Oliwia Gołyga[2], Mateusz Słowikowski[2,5], Jacek Kacperski[2], Marcin Siekacz[2], Grzegorz Muzioł[2], Piotr Nowicki[6], Aleksandr Kazakov[7], Jerzy Wróbel[6], Tomasz Czyszanowski[8], and Jan Suffczyński*[1]

[1]Institute of Experimental Physics, Faculty of Physics, University of Warsaw, 5 Pasteura St., 02-093 Warsaw, Poland

[2]Institute of High Pressure Physics, Polish Academy of Sciences, 29/37 Sokolowska St., 01-142 Warsaw, Poland

[3]CENTERA labs, Institute of High Pressure Physics, Polish Academy of Sciences, 29/37 Sokolowska St., 01-142 Warsaw, Poland

[4]CENTERA, CEZAMAT, Warsaw University of Technology, 19 Poleczki St., 02-822 Warsaw, Poland

[5]CEZAMAT, Warsaw University of Technology, 19 Poleczki St., 02-822 Warsaw, Poland

[6]Institute of Physics, Polish Academy of Sciences, 32/46 Lotnikow Av., 02-668 Warsaw, Poland

[7]International Research Centre MagTop, Institute of Physics, Polish Academy of Sciences, 32/46 Lotnikow Av., 02-668 Warsaw, Poland

[8]Institute of Physics, Łódź University of Technology, 217/221 Wólczańska St., 90-451 Łódź, Poland

*Email: Jan.Suffczynski@fuw.edu.pl

Abstract

This file contains supporting information to the article "Blue lasing at room temperature based on a quasi-bound state in the continuum".

S1 Numerical simulations and design of the structures

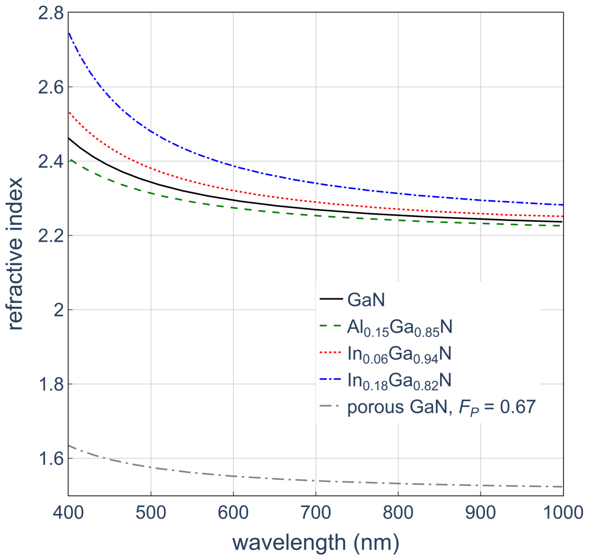

Figure S1: Refractive index as a function of the light wavelength for GaN, nanoporous GaN with porosity of 0.67, (Al,Ga)N, and (In,Ga)N used in the numerical simulations of the studied GaN-based subwavelength grating structures.

The studied structures comprising GaN-based subwavelength gratings are designed by using numerical calculations employing Plane-Wave Admittance Method (PWAM).[1] The PWAM provides an efficient numerical solution of Maxwell's equations for layered dielectric structures. In the simulations, we operate on

tensorial permittivities, what allows us to include anisotropy. As a result of the calculations, we obtain the energy, quality factor, and electric field distribution of the optical modes of the structure.

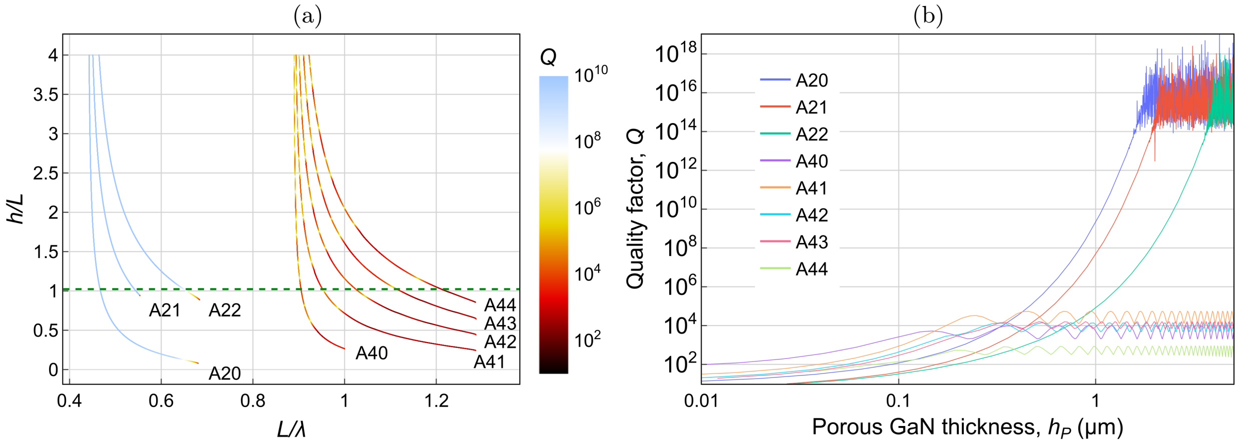

Figure S2: (a) Calculated quality factor Q of the optical modes of the GaN-based subwavelength grating as a function of normalized height h/L and normalized period L/λ of the grating for fill factor $F = 78\%$. The green dashed line indicates the case of $h = 510\,\text{nm}$ and $L = 500\,\text{nm}$, *i.e.* the chosen passive design. (b) Quality factor Q of the selected modes of the passive structure as a function of the thickness of the porous GaN layer h_P with porosity $F_P = 0.75$ and grating's geometry parameters fixed at: period $L = 500\,\text{nm}$, depth of etching $h = 510\,\text{nm}$, width of etched stripes $L(1 - F) = 112\,\text{nm}$.

We start with calculating of quality factor Q of the optical modes of the GaN-based subwavelength grating as a function of the height h and grating period L normalized to the wavelength of the light λ. We consider a single period of the grating with periodic boundary conditions in the y direction, infinite extent in the x direction, sandwiched between infinitely high air and GaN layers in the z direction. Supp. Fig. S1 presents dispersion curves of the refractive index for the materials employed in the present work in the numerical simulations of the studied GaN-based subwavelength grating structures. We use the dispersion function of the refractive index of solid GaN taken from Ref. [2], AlGaN – from Ref. [3], and InGaN – from Ref. [4]. We assume that nanoporous GaN layer of thickness h_p has the real part of the refractive index of $n_P(\lambda) = \sqrt{(1 - F_P) \cdot n^2_{\text{GaN}}(\lambda) + F_P}$, where F_P is the porosity of the layer and $n_{\text{GaN}}(\lambda)$ – wavelength-dependent, real refractive index of solid GaN.[5] The quality factor of the SWG modes are determined from the formula: $Q = -0.5\lambda_{re}/\lambda_{im}$, where λ_{re} and λ_{im} are the real and imaginary parts of the complex wavelength, respectively.

Since nanoporous layers may exhibit anisotropy of the refractive index[6, 7], we perform calculations independently changing components of the permittivity tensor. Based on the results of the calculations (see Supp. Fig. S4), we state that only the permittivity in the direction parallel to the SWG stripes affects the Q factor in zero angle.

The Supp. Fig. S2a shows the results of the calculations performed for the passive structure with the assumption that the grating fill factor F is 78%. As can be seen, the optical modes of the SWG with the highest Q can be divided into two groups. For the L/λ below 0.65, thus in the subwavelength regime in the air and in the substrate, the Q is of the order of $10^8 - 10^{10}$ practically independently of the grating height. This suggests that these modes are strictly BIC.

For the L/λ above 0.65, thus when the subwavelength condition in the substrate is no longer met, the Q of the SWG can still reach the order of 10^6. It can be observed that the larger h, the larger Q. These modes are expected to be of a quasi-BIC type.

In order to highlight the role of the nanoporous GaN layer and to determine the optimal design of the structures studied, we calculate the quality factor of the SWG as a function of the nanoporous GaN thickness h_P, as shown in Supp. Fig. S2b. We can see that BICs can be present in the structure upon the condition that the h_P exceeds 1.1 μm. For the quasi-BIC, the requirement for the quality factor is relaxed, and h_P should exceed several hundred nanometers.

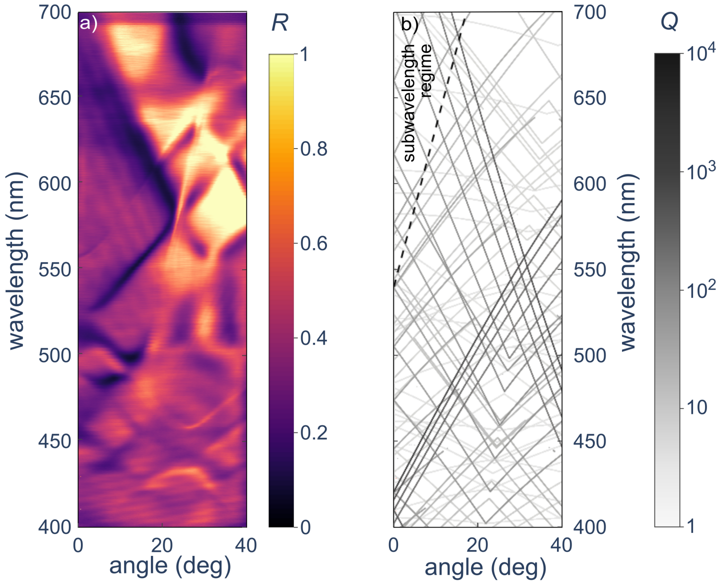

Figure S3: a) Experimentally obtained angle-resolved reflectivity map of the passive structure in TE polarization, b) numerically calculated dispersion curves of modes confined in the nanoporous layer. The color intensity of the curves encodes the calculated Q-factor value. A black dashed line indicates the cut-off of the first diffraction order of the grating.

As Supp. Fig. S4 shows, the dependence of the Q factor vs porosity is not monotonic. Anyway, the general trend is evident. The Q increases with porosity, thus the degree of the decoupling of the SWG modes from the solid GaN substrate. In particular, the consecutive maxima found for porosity of 0.62, 0.81 and 0.93, Q factor reach values of $1.1 \cdot 10^4$, $2.1 \cdot 10^4$ and $6 \cdot 10^4$, respectively.

The above results show that both, the nanoporous GaN layer thickness and porosity, strongly impact the optical performance of the GaN-based subwavelength grating that is deposited on this layer.

In order to determine the impact of the sidewall roughness of a stripe in the GaN-based subwavelength grating on the quality factor Q of the optical modes confined in the grating, we calculate Q as a function of the roughness parameter R_q. The results of the calculations are shown in Supp. Fig. S5. As it can be seen, the Q decreases with the increasing roughness, as expected.

Based on SEM images we determine the R_q of the fabricated GaN-based gratings in the passive structure. As shown in Supp. Sec. S2, the R_q before the smoothening using TMAH remains in the approximate range of 4.6–4.9 nm. It decreases approximately to 3.6–3.85 nm after the smoothening. As a result, the Q increases by the respective factor of around 1.4–1.8, depending on the mode of the grating. The Q enhancement is the largest for the modes with the largest intensity of the electric field on the edge of the stripe, as these modes are the most susceptible to the roughness-induced scattering.

S2 Structures fabrication

S2.1 Epitaxial growth of the samples

The samples studied are grown by plasma-assisted molecular beam epitaxy (PAMBE) in a custom-designed VG90 Semicon MBE reactor, as described in the main text. The epitaxial growth is carried out under metal-rich conditions. The growth temperature and presence of the metallic layer during the epitaxy are controlled by laser reflectometry. Commercially available bulk GaN substrates with a threading dislocation density of $5 \cdot 10^6$ cm^{-2} are used. Supp. Tabs. S1 and S2 provide the geometry details of the passive and the active structure, respectively.

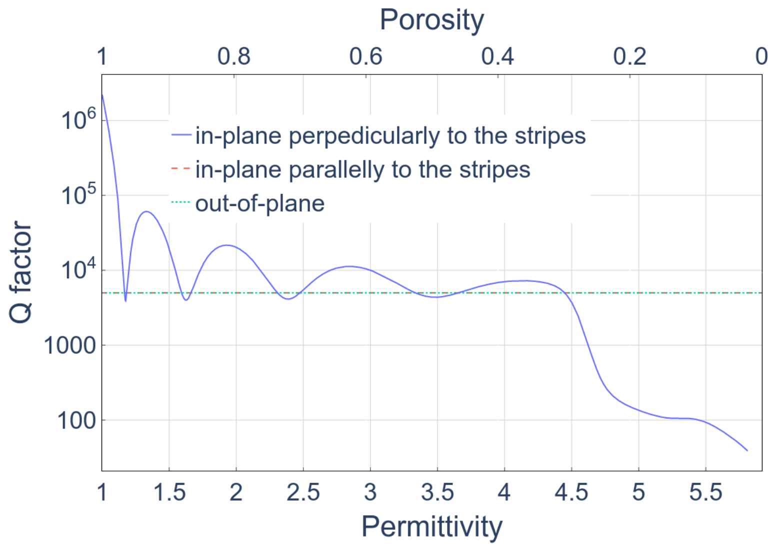

Figure S4: Quality factor Q of the lasing A43 mode of the active structure (comprising GaN-based subwavelength grating with (In,Ga)N QWs and a porous GaN layer underneath). The Q is plotted as a function of the permittivity (bottom horizontal axis) or porosity (top horizontal axis) of the porous layer in selected spatial directions.

**Thickness (nm)**	**Material**
505	GaN
2000	GaN:Si (Si: $3 \cdot 10^{19}$ cm^{-3})
50	GaN
50	GaN:Si (Si: $2 \cdot 10^{18}$ cm^{-3})
	GaN substrate

Table S1: Epitaxial structure of the passive design. The GaN-based grating is etched out of the top GaN layer. The nanoporous GaN layer is formed out of the 2000 nm thick GaN:Si layer.

S2.2 GaN-based subwavelength grating fabrication by electron-beam lithography

After the plasma assisted MBE growth, the sample surface is cleaned in HCl solution to remove any metal residues. Before the electronolitography process, the sample was additionally cleaned in acetone for 10 min at 60°C and then in isopropanol for 5 min at 60 °C before a resist deposition. After drying with nitrogen, the surface is covered with 120 nm of PMMA 950 K (3%) positive resist by spin-coating (4000 rpm, 60 s). Next, the resist is baked on a hotplate at 180°C for 30 min. Since unintentionally doped GaN epi-layers are not highly conductive, the resist is additionally covered with a conductive polymer, Electra (AR-PC 5090.02 by AllResist), and baked on a hotplate at 105°C for 5 min. We find that a 60 nm polymer layer is optimal for eliminating charge accumulation.

For electron-beam exposure on the passive structure we use the scanning electron microscope (SEM) JEOL JSM-6400 coupled with the RAITH Elphy lithography system, while for the active structure, we use JEOL JBX-9300FS. For development, the solution of distilled water and isopropanol (H_2O:IPA, 3:7) is used for 30 seconds, followed by a rinse in distilled water for 2 times 15 seconds. Finally, the 20 nm thick nickel layer is sputtered onto the prepared surface to serve as an etching mask. The lift-off process starts with placing the sample in hot acetone for one hour, then immersing it in an ultrasonic bath for 30 minutes. For the passive structure, 20 nm thick Ni mask is deposited and an e-beam patterning is executed with an acceleration voltage of 30 kV, 100 pA beam current, and doses varied from 180 mC/cm^2 to 240 mC/cm^2. For the active structure, Ni mask is 15 nm thick and respective parameters are: 100 kV, 1 nA and 190 µC/cm^2. High-resolution e-beam resist and conductive protective coating are applied. A proximity effect correction

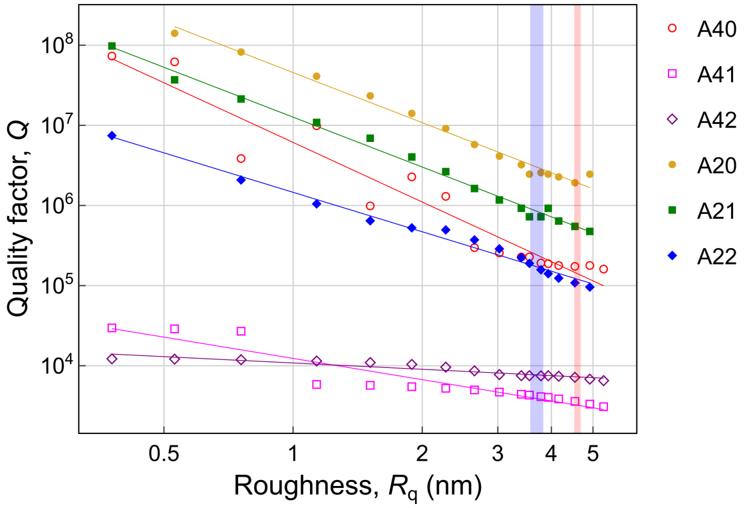

Figure S5: Quality factor Q of the optical modes confined in the GaN-based subwavelength grating in the passive structure with period $L = 500\,\text{nm}$, and fill factor $F = 0.736$ obtained from simulations as a function of roughness parameter R_q. The calculated values are represented as points. Lines are guides to the eye. Reddened and blued areas indicate a region of roughness of the passive structures before and after TMAH treatment, as determined from SEM images, respectively.

algorithm is used to optimize the exposure dose with respect to the pattern geometry.

In the final step, the grating pattern is dry etched by RIE in a mixture of chlorine (Cl_2) and argon (Ar). We use the Plasmalab Sytem100 from Oxford Instruments Plasma Technology. The groove depth is 505 nm for the passive structure. For the active structure the final groove depth is found to depend on the groove width. The groove depth is 225, 250, and 275 nm ±10 nm for the nominal groove widths of 80, 89, and 100 nm, respectively. Note that the actual fabricated groove width as measured on SEM images is slightly narrower than the nominal ones and it is 75, 85 and 90 ±3 nm, for nominal of 80, 89, and 100 nm, respectively. RIE has a parallel plate chamber configuration with an additional ICP plasma source. For the RIE process, the sample is attached to a 2" silicon carrier wafer using hot melt glue (Crystalbond). This is necessary to better control the temperature of the sample during RIE etching and thus, to have a stable etch rate. Etching parameters are as follows: pressure 15 mT, RF power 20 W, ICP power 200 W, Cl_2 flow 24 scmm, Ar flow 8 sccm, table temperature 15°C. Etch rate and etch depth are controlled by using of a laser reflectometer. The etch rate is kept at 110 nm/min.

S2.3 Smoothening of sidewalls of GaN-based subwavelength grating

The orientation of the gratings with respect to the crystallographic directions of the GaN substrate is intentionally selected in such a way that the stripes are perpendicular to the <1-100> direction of bulk GaN, which is commonly called the m direction. It allows us to introduce a processing step dedicated to improving the sidewall surface smoothness, namely, etching in a TMAH solution. The TMAH treatment is done for 1 min at 80°C to maintain the lowest possible groove width. The comparison of the gratings top-view before and after TMAH treatments is shown in Figure 2 in the main text. The groove width in this representative case increased from 95 nm to 115 nm as a result of the TMAH treatment.

S2.4 Fabrication of a nanoporous GaN layer via electrochemical etching

To form a nanoporous layer beneath the subwavelength gratings, the samples are electrochemically etched in 0.3M oxalic acid in a three-electrode cell described elsewhere.[8] High n-type doping [Si] $3 \cdot 10^{19}$ cm^{-3} is essential to achieve controllable porosity and a high pore density. The passive structure was etched at 7 V for 20 h, while the active structure was etched at 7 V for 90 min. Etching of the GaN:Si layer beneath the gratings is verified using an optical microscope. In the case of the SWG in the active structure, etching

Thickness (nm)	Material	Role
400	GaN	
20	$Al_{0.15}Ga_{0.85}N$	
64	$In_{0.06}Ga_{0.94}N$	
10.4	$In_{0.18}Ga_{0.82}N$	Quantum Well
150	$In_{0.06}Ga_{0.94}N$	
10.4	$In_{0.18}Ga_{0.82}N$	Quantum Well
20	$In_{0.06}Ga_{0.94}N$	
5	GaN	
600	GaN:Si (Si: $3 \cdot 10^{19}$ cm^{-3})	
50	GaN	
50	GaN:Si (Si: $2 \cdot 10^{18}$ cm^{-3})	current spreading layer
	GaN	substrate

Table S2: Epitaxial structure of the active design. The GaN-based grating is etched out of the top GaN layer. The nanoporous GaN layer is formed out of the 600 nm thick GaN:Si layer.

proceeds up to 39 µm from the grooves. The cross-sections of the samples with subwavelength grating on porous GaN and on InGaN QW are presented in Figure 2c–d. Degree of porosity in the case of both, passive and active, structures is around $F_P = 0.7$.

Optical microscope images of the InGaN-based subwavelength grating are shown in Supp. Figure S6. A small magnification view in Supp. Figure S6a presents an array of gratings with different periods and groove widths. Designed periods ranged from 440 to 470 nm and three nominal groove widths are chosen: 80, 89, and 100 nm. Every grating is fabricated in four nominally equivalent copies (four columns in Supp. Figure S6a). The magnified view presented in Supp. Figure S6b shows the details of the islands with the gratings that are separated by 5-µm-wide grooves. The grating area is marked with a pink dashed line. Lateral electrochemical etching proceeds from the sidewall of the separating grooves towards the middle of an island over a distance of approx. 39 µm. Respective SEM top-view image showing more details of the grating after TMAH sidewall smoothening and ECE is presented in Supp. Fig. S6c. The sample is cleaved to assess the grating geometry. The direction of the grating cross-section is marked schematically by yellow dashed line. The respective SEM image of the grating cross-section is presented in Supp. Fig. S6d. Note the crystallographic orientation marked in the images.

S3 Reflectivity measurements

The schematics of the experimental setup is presented in Supp. Figure S7. A Hamamatsu Energtiq EQ-99X white lamp is a light source. The beam emitted by the lamp is collimated and directed towards a nonpolarizing 50/50 beamsplitter (NP BS). After passing through the NP BS, it is directed towards a microscope objective with NA = 0.75 and focused to a few µm spot on the sample surface. The reflectivity signal emerging from the sample collected by with the same objective is reflected by the NP BS. Next, it passes through the first lens, which performs a Fourier transform on the signal, converting a 2D real space image into a photon in-plane momentum one. The second lens ensures that the magnification of the momentum space image is adjusted to the size of the matrix of a CCD camera placed on the output of the spectrometer. The spectrometer of length 300 mm is equipped with a diffraction grating of 1800 gr/mm. Also this lens is placed on automated XY stages that allow it to move (with a step of 50 µm) in two directions perpendicular to the optical axis, enabling registration of a full topographic image of the photon in-plane momentum space. The beam then passes through half-wave plate and a linear polarizer and enters the spectrometer through a slit (width 100 µm). With the slit, we select a slice through the momentum space. This slice is then diffracted on a grating and projected onto a CCD camera, producing an image like the one shown in Figure 3a.

Thus, upon entering the spectrometer, a large fraction of the beam cross-section is rejected, and only its narrow slice is recorded by the CCD camera. This slice encodes the dispersion relation of a particular, say k_x wavevector. The perpendicular wavevector, k_y is not projected; only a constant value of this vector

is selected by the slit. Thus, an image registered by the CCD camera, such as shown in Figure 3a is a projection of k_x wave-vector for a fixed value of k_y vector. Moving sideways the second lens of the Fourier imaging set shifts the position of the beam on the slit. This changes the value of k_y for which the image is detected. By taking a series of images at consecutive lens positions, from one edge of the beam to the other, we obtain the full 3D tomography of the photon energy *vs* k_x and k_y dependence.

A polarization resolved reflectivity measurement on the GaN-based SWG in the passive structure allows us to reveal a polarization vortex in the vicinity of the quasi-BIC at $k = 0$, as shown in Supp. Fig. S8. The detection is conducted in four selected linear polarizations: horizontal, vertical, diagonal, and anti-diagonal. This gives us components of the Stokes vector of the reflected light, and finally, its polarization vector angle.[9] The presence of the vortex provides proof of quasi-BIC existence in the studied structures.

S4 Photoluminescence measurements

Emission spectra of the active structure for consecutively increasing excitation power density are shown in Supp. Fig. S9. The emission wavelength is around 445 nm. In general, the tuning of the lasing wavelength via geometry is restricted by the gain spectrum of the active region of the structure. While extending the grating period range could potentially broaden the tuning window, the lasing wavelength must align with the QW gain spectrum. By increasing the In content in the QW, we can tune the emission from around 360 nm to around 450 nm (as in the present work, where a structure containing two high-quality 10.4 nm wide $In_{0.18}Ga_{0.82}N$ QWs is studied) or higher. The exact lasing wavelength is then tuned via the geometry of the SWG – we vary the grating period and trench width, both of the order of 100 nm, with a resolution better than 10 nm. Material constraints impose additional practical limits on extending the BIC lasing wavelength towards both long and short wavelengths. Current epitaxial techniques (MBE, as used in this work, and MOCVD) reliably produce high-quality InGaN QWs with indium compositions up to approximately 25%, enabling extended tuning toward longer wavelengths. Conversely, achieving shorter wavelengths using GaN or AlGaN QWs is feasible in principle, but introduces fabrication challenges: the correspondingly smaller grating features require pushing the spatial resolution limits of electron beam lithography patterning and strict control of the dry-etching process. Thus, the optimal design strategy depends on balancing the target wavelength range against the available material quality and lithographic capabilities.

To verify the uniformity of the grating, the emission from a representative grating is collected at 64 positions arranged in an 8×8 rectangular grid, ensuring that the spacing between points exceeds the excitation spot size. The intensity ratio of TE- to TM-polarized emission, based on statistics from 59 points, is around 4.0 ± 0.3. (5 points out of 69 have been disregarded as coming from an area in the center of the grating where nanoporous layer is not etched.) As Supp. Fig. S10 b-d shows, emission parameters such as intensity, spectral position, and linewidth show only a small spread of their values. This indicates that the grating has a uniform structure.

References

1. Dems, M., Kotynski, R. & Panajotov, K. PlaneWave Admittance Method — a novel approach for determining the electromagnetic modes in photonic structures. *Opt. Express* **13,** 3196–3207 (2005).
2. Barker Jr, A. & Ilegems, M. Infrared lattice vibrations and free-electron dispersion in GaN. *Physical Review B* **7,** 743 (1973).
3. Bergmann, M. & Casey Jr, H. Optical-field calculations for lossy multiple-layer Al x Ga 1- x N/In x Ga 1- x N laser diodes. *Journal of Applied Physics* **84,** 1196–1203 (1998).
4. Muzioł, G. *et al.* Waveguide design for long wavelength InGaN based laser diodes. *Acta Physica Polonica A* **122,** 1031–1033 (2012).
5. Braun, M. M. & Pilon, L. Effective optical properties of non-absorbing nanoporous thin films. *Thin Solid Films* **496,** 505–514 (2006).
6. Griffin, P. H. & Oliver, R. A. Porous nitride semiconductors reviewed. *Journal of Physics D: Applied Physics* **53,** 383002 (2020).

7. Elafandy, R. T. *et al.* Study and application of birefringent nanoporous GaN in the polarization control of blue vertical-cavity surface-emitting lasers. *ACS Photonics* **8,** 1041–1047 (2021).

8. Sawicka, M. *et al.* Revealing inhomogeneous Si incorporation into GaN at the nanometer scale by electrochemical etching. *Nanoscale* **12,** 6137–6143 (10 2020).

9. Pruszyńska-Karbownik, E. *et al.* Optical Bound States in the Continuum in Subwavelength Gratings Made of an Epitaxial van der Waals Material. *ACS Nano* **20,** 7426–7437 (2026).

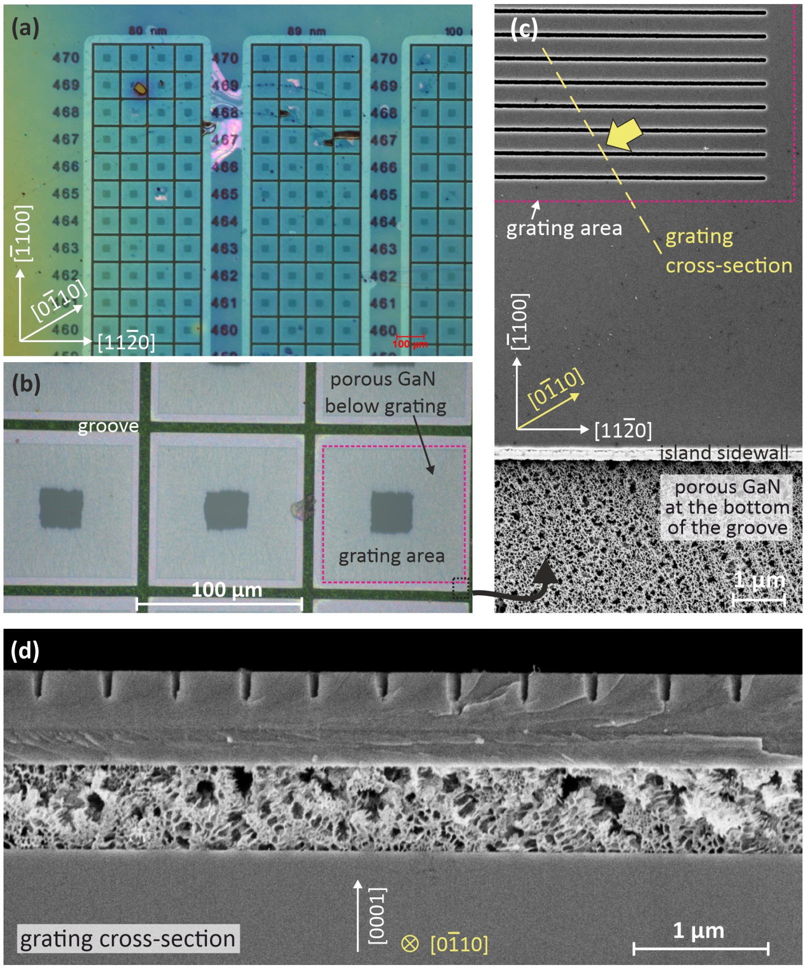

Figure S6: (a) Top-view optical microscope image of an array of the subwavelength gratings in the active structure after electrochemical etching. Gratings have periods from 440 to 470 nm and nominal groove widths of 80, 89 and 100 nm. (b) Magnified optical microscope image of an exemplary 95 $\times$ 95 µm^2 island with a grating formed over the area of 85 $\times$ 85 µm^2, separated by 5-µm-wide grooves. Porous GaN below the grating is formed by lateral electrochemical etching from the sides of the island and is visible as brighter than the middle region, which is left non-porous. (c) Top-view SEM image of a grating. A yellow dashed line indicates the direction of the cross-section through the grating. (d) Cross-section of an exemplary grating viewed along the [0$\bar{1}$10] direction. The crystallographic orientation is marked on the images.

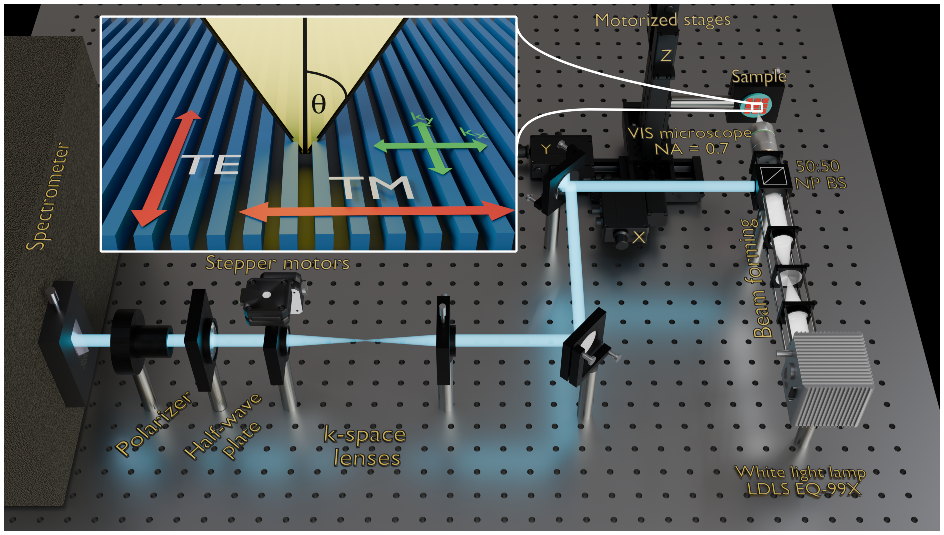

Figure S7: Schematic of the setup for reflectivity measurements. The setup enables angularly resolved detection, that is with the resolution in k-space (photon in-plane momentum space). The inset shows the directions of electric field oscillation for TE and TM polarized light relative to the grating.

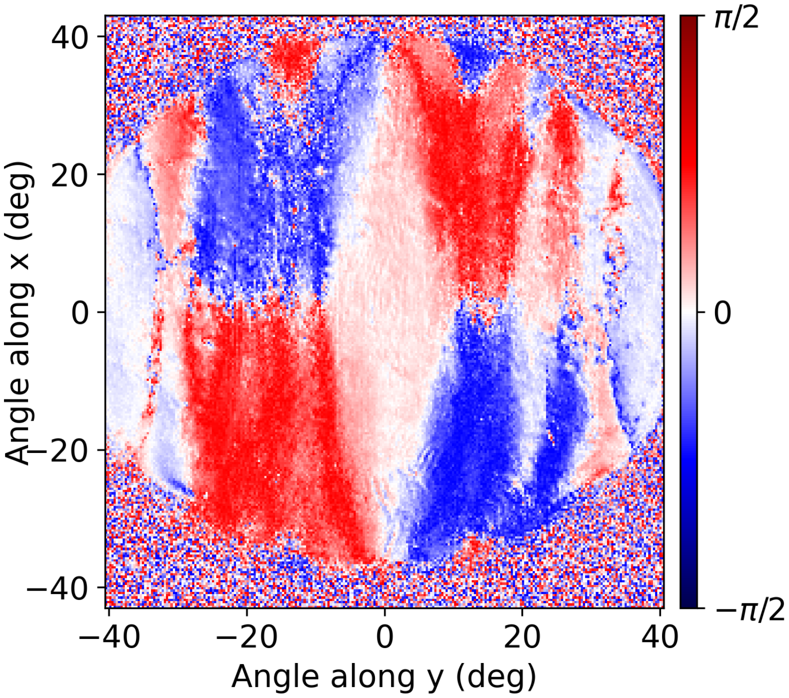

Figure S8: A polarization vortex in the vicinity of the quasi-BIC at 539 nm at $k = 0$ detected in polarization resolved reflectivity of the GaN-based SWG in the passive structure. The SWG period 500 nm, etching depth 510 nm, and fill factor 0.78.

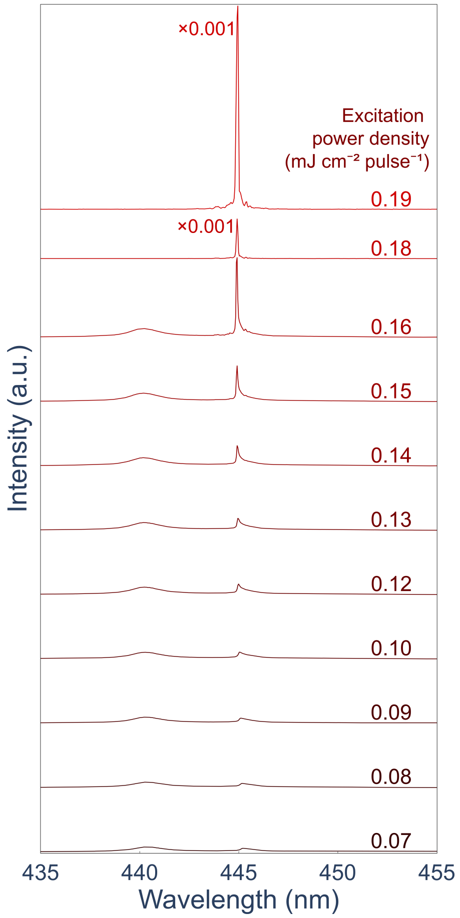

Figure S9: Angle-integrated emission spectra of the active structure for consecutively increasing excitation power density. The sample is pulsed-excited at 375 nm at 300 K. The lasing threshold is 0.16 mJ cm^{-2} $pulse^{-1}$. The intensity of the spectra over the threshold is divided by a factor of 1000 for clarity.

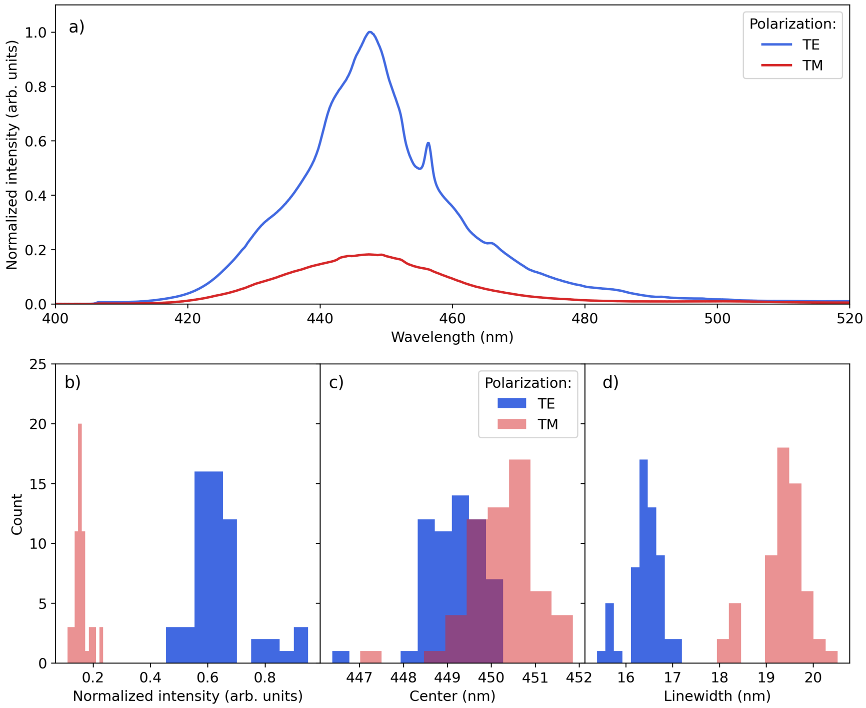

Figure S10: Statistics of the emission from 59 different points of an exemplary grating in the active structure. a) Emission in TE and TM polarization integrated over all detectable emission angles. TE to TM intensity ratio attains around 4. b) Statistics of the integrated emission intensity for TE and TM polarization normalized to the maximum value out of the entire set. c) Statistics of the emission centroid for TE and TM polarization. d) Spectral linewidth of the integrated emission for TE and TM polarization.

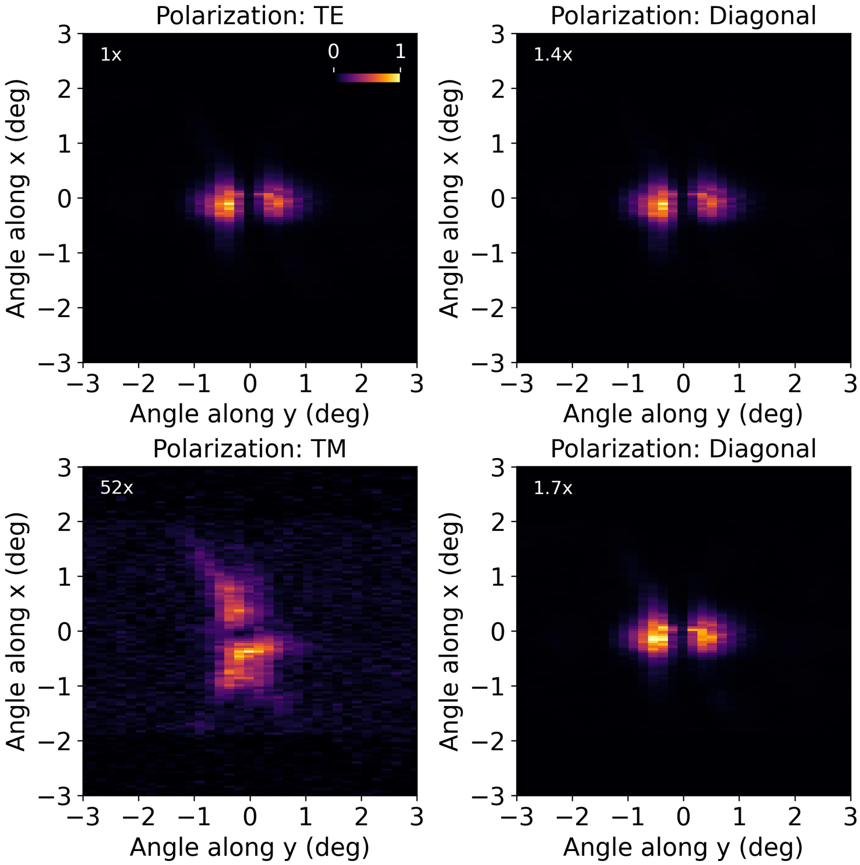

Figure S11: Polarization rotation around a quasi-BIC mode at 444.9 nm observed in the lasing emission (the intensity of the signal is normalized to its maximum value for a given linear polarization of detection, independently for each panel). The change of the detected linear polarization of emission reveals rotation of the emission direction around $k = 0$. This confirms that the emission takes place from around a BIC state. The small difference in polarization angles in the vortex stems from the anisotropy of the structure; the grating structure has a lowered rotational symmetry, which results in TE polarization being the dominant one, around 52 times stronger than the TM polarization.
